# The effects of losartan on cytomegalovirus infection in human trabecular meshwork cells

**DOI:** 10.1371/journal.pone.0218471

**Published:** 2019-06-19

**Authors:** Jin A. Choi, Ju-Eun Kim, Hyun-hee Ju, Jiyoung Lee, Donghyun Jee, Chan Kee Park, Soon-young Paik

**Affiliations:** 1 Department of Ophthalmology, College of Medicine, St. Vincent’s Hospital, The Catholic University of Korea, Seoul, Republic of Korea; 2 Department of Microbiology, College of Medicine, The Catholic University of Korea, Seoul, Republic of Korea; 3 Department of Ophthalmology, College of Medicine, Seoul St. Mary’s Hospital, The Catholic University of Korea, Seoul, Republic of Korea; Max Delbruck Centrum fur Molekulare Medizin Berlin Buch, GERMANY

## Abstract

**Background:**

Human cytomegalovirus (CMV) has been emerged as one of the causes of acute recurrent or chronic hypertensive anterior uveitis in immunocompetent. In hypertensive anterior uveitis, human trabecular meshwork (TM) cells are considered a focus of inflammation. We investigated the effects of losartan, a selective angiotensin II receptor antagonist, on CMV infection in human TM cells.

**Methods:**

Human TM cells were infected with CMV AD169. Virus infected and mock-infected cells were treated with losartan or dexamethasone or ganciclovir with or without transforming growth factor (TGF)-β1. Viral DNA accumulation and host cell response were analyzed using real-time PCR. Levels of secreted TGF-β1 were measured by determining its concentration in conditioned medium using a commercially available sandwich enzyme-linked immunosorbent assay (ELISA) kits.

**Results:**

CMV infection significantly increased the concentrations of the secreted TGF-β1 at 3, 5, and 7 day post infection in TM cells. Treatment with dexamethasone or losartan significantly decreased the levels of TGF-β1, whereas treatment with ganciclovir did not affect TGF-β1 levels. TM cells treated with TGF-β1 along with the presence of losartan for 48 hours showed marked decrease in the expression of α-smooth muscle actin (SMA), lysyl oxidase (LOX), connective tissue growth factor (CTGF), fibronectin and collagen-1A, compared with cells treated with TGF-β1 alone. CMV-infected TM cells stimulated by TGF-β1 significantly increased the expression of α-SMA and CTGF, which were attenuated by additional treatment with losartan.

**Conclusion:**

Losartan inhibited the expression of TGF-β1 and fibrogenic molecules in human TM cells. Thus, losartan has the potential to decrease TM fibrosis in patients with CMV-induced hypertensive anterior uveitis.

## Introduction

Anterior uveitis, the most common type of intraocular inflammation, is the most commonly associated with the elevation of intraocular pressure (IOP). The etiology of anterior uveitis includes infectious, non-infectious, and secondary origin to masquerade syndrome. The most common form of anterior uveitis is HLA-B27 associated uveitis, in which IOP is often reduced. Contrary to the HLA-B27 associated uveitis, anterior uveitis secondary to virus infection is characterized by the elevation of IOP at the time of inflammation. The three main herpes viruses, herpes simplex virus (HSV)-1, varicella zoster virus, and CMV have been focused as a cause of anterior uveitis [1].

Among the herpes viruses, CMV has been increasingly recognized as a cause of acute recurrent or chronic anterior uveitis associated with ocular hypertension or corneal endotheliitis in immunocompetent patients [2, 3]. High CMV viral loads have been correlated with number of recurrences and corneal endothelial damage [4, 5]. Longer duration of uveitis and frequent relapses lead to glaucomatous damage in 24–26% of cases [6]. Among the forms of viral anterior uveitis, CMV anterior uveitis is known to accompany a higher number of eyes requiring glaucoma filtering surgery and severe corneal endothelial cell loss compared with CMV-negative cases [7]. The potentially vision-threatening complications of the CMV anterior uveitis may attribute to the intrinsic characteristics of the pathogen. The antivirals routinely used for HSV and VZV do not treat CMV anterior uveitis. In addition, the systemic anti-CMV agent carries systemic side effects requiring routine lab monitoring, which hinders long term systemic anti-viral prophylaxis in CMV anterior uveitis.

In many CMV uveitis cases, there is generally a clinical response to topical steroid and anti-CMV agents, only to recur with the cessation of treatment [3, 8]. The development of novel disease-modifying drugs based on the pathogenic mechanism is therefore necessary for more effective treatment of CMV anterior uveitis.

CMV infection is associated with many fibrotic diseases such as congenital hepatic fibrosis, idiopathic pulmonary fibrosis, enhanced chronic renal allograft rejection, and idiopathic pulmonary fibrosis [9–11]. Transforming growth factor (TGF)-β1, a fibrogenic cytokine, is highly expressed in CMV-infected renal allografts [12]. CMV infection induces TGF-β1 secretion in renal epithelial cells, astrocytes, osteosarcoma cells, and fibroblasts *in vitro* [13–15]. In a previous study, we found that CMV successfully replicated and enhanced TGF-β1 production in human trabecular meshwork (TM) cells [16], the key cell type regulating IOP [17]. In addition, CMV infection is thought to aggravate fibrosis through the activation of TGF-β1 [11].

TGF-β is increased in the aqueous humor in glaucoma patients and is one of the major molecular signatures of this disorder [18]. Increased TGF-β induces pro-fibrotic signaling, ultimately resulting in accelerated accumulation of extracellular matrix (ECM) and stiffening of the TM cells, which leads to an increase in outflow resistance [17]. In the modulation of the ECM outflow pathway, the ocular renin-angiotensin system (RAS) has been thought to be a potential therapeutic target [19–21]. RAS systemically regulates blood pressure homeostasis, systemic fluid volume, and electrolyte balance. In addition to these obligatory roles, angiotensin II regulates fibrosis, inflammation, proliferation, and vasoconstriction. RAS inhibitors have been successfully used in the treatment of cardiovascular diseases involving extensive ECM remodeling in the myocardium and in fibrotic conditions affecting the kidneys [22, 23]. As a paracrine system, RAS components and their downstream targets are known to exist in human TM cells [19]. Based on the pro-fibrotic changes induced by CMV infection, we hypothesized that the inhibition of RAS in human TM cells may have therapeutic benefit in patients with CMV-induced hypertensive anterior uveitis. Considering the role of TM cells in the regulation of IOP, a model of CMV infection in human TM cells could be a useful tool to test the pathogenic mechanism of CMV hypertensive anterior uveitis. In the present study, we investigated the effects of losartan, a selective angiotensin II type 1 receptor (AT1R) inhibitor in association with CMV-induced changes in TM cells, and compared its effects with those of current treatment strategies, including steroid and anti-viral agent.

## Methods

### Materials

The following reagents were obtained from the respective commercial vendors. Recombinant human TGF-β1 from R&D Systems (Minneapolis, MN, USA), losartan from Selleck Chemicals (Houston, TX, USA), dexamethasone from Sigma-Aldrich (St. Louis, MO, USA), ganciclovir from Selleck Chemicals, normal horse serum (RTU Vectastain Universal Elite ABC Kit) from Vector Laboratories (Burlingame, CA, USA), murine monoclonal anti-CMV IE antibody (anti-CMV Immediate Early Antigen Antibody, #LS-C103255) from LSBio (Seattle, WA, USA), a goat anti-mouse antibody (VectaFluor R.T.U. DyLight 488 anti-mouse) from Vector Laboratories, rhodamine phalloidin and Vecta-Stain mounting media (VECTASHIELD) from Invitrogen (Carlsbad, CA, USA), a TGF-β1 enzyme-linked immunosorbent assay (ELISA) kit (Total TGF-β1 ELISA Kit with precoated plates) from BioLegend (San Diego, CA, USA), and a MTS assay kit (CellTiter 96^®^ AQueous One Solution Cell Proliferation Assay) from Promega (Medison, WI, USA).

### Cells

Human TM cells were derived from two separate donors aged 39 years (male) and 16 years (female) without any known ocular diseases. Informed consent for tissue donation was obtained from the patients or their relatives (from a parent in case of 16 years old donor), and the study protocols were approved by the Institutional Review Board at the Catholic University of Korea in accordance with the Declaration of Helsinki for experiments involving human tissues and samples. The TM tissues were dissected and cultured as previously described [24]. The maintenance growth medium contained low glucose Dulbecco’s Modified Eagle’s Medium (DMEM) supplemented with 15% fetal bovine serum (Invitrogen-Gibco, Grand Island, NY, USA), 1% penicillin-streptomycin, and fibroblast growth factor-2 (1 ng/mL). For repeated experiments, primary TM cells obtained from ScienCell Research Labs (Carlsbad, CA, USA) were used and cultured to 100% confluence in Trabecular Meshwork Cell Medium (ScienCell Research Labs) [25]. The TM cells in passage 4–6 were seeded into 6 well plates until the cells reached confluency.

### Viruses

Human foreskin fibroblasts (HFF) were used to propagate viral stocks. For purification, the cells were treated by freezing and thawing once, then centrifuged at 2,000 rpm for 20 min. Supernatant fluids were used as virus inoculum. Cell-free CMV was collected by filtration of the infected cell medium or extracted through a 0.45 μm filter, and loaded on a 10–55% sucrose gradient and centrifuged at 20,000 rpm for 1 h. The CMV pellet was washed and suspended in 2 ml of DMEM / 2% fetal bovine serum and was stored in aliquots at -80°C until use. Virus stocks were titrated by using a 50% tissue culture infectious dose (TCID_50_) assay on HFFs, using the method of Reed and Muench. When TM cells reached confluence, they were incubated with the virus stock preparation for a 2-h adsorption period at 37°C in 5% CO_2_ with a multiplicity of infection (MOI) of 1 and 0.1. After removal of the viral inoculum, the infected cells were washed once with 1× phosphate-buffered saline. All cell culture experiments were performed after serum starvation.

### Viral DNA replication assays

TM cells seeded onto 6 well plates (4 x 10^5^ cells/well), and infected with CMV AD169 at MOI of 1 were treated with dexamethasone at 100 nM or ganciclovir at 10, 100 μM, or losartan at 1, 10 μM for 1, 3, 5 and 7 days, respectively. Human CMV capsids were pelleted from culture supernatants at 1, 3, 5, and 7 days post infection (dpi) and then resuspended and treated with DNase I. Capsids were disrupted using a Qiagen column (QIAmp DNA Mini Kit; Qiagen, Hilden, Germany), and DNA was quantitated by real-time quantitative PCR using nucleotide primers proven specific for the *AD169 UL26* gene [26]. Real-time PCR with β-actin primers was also performed to serve as an internal control for input DNA.

### Cell viability assay

The effects of dexamethasone, losartan, and ganciclovir on viability in cultured TM cells were evaluated using MTS assays. Cells were seeded at their optimal cell density (1×10^4^ cells/well) into a 96-well microtiter plate and were incubated overnight to allow cell attachment. Cells were treated with various concentrations of dexamethasone or losartan, or ganciclovir at 37°C under 5% CO_2_ for 1, 3, 5, and 7 days, respectively. At the end of each incubation period, the cell viability was determined according to the manufacturer's instructions.

### Elisa for TGF-β1

TM cells were grown in 6 wells plates, either untreated or infected with CMV AD169 at a MOI of 1 and/or incubated with dexamethasone at 100 nM or ganciclovir at 10, 100 μM, or losartan at 1, 10 μM for 1, 3, 5 and 7 days, respectively. The level of secreted TGF-β1 was measured by determining its concentration in conditioned medium using a commercially available sandwich ELISA kit at 1, 3, 5, and 7 dpi. Conditioned medium was harvested and cleared by centrifugation, then stored at −70°C. Conditioned medium was acid-activated and directly assayed using an ELISA plate reader at 450 nm according to the manufacturer’s instructions. Protein concentrations were calculated from a standard curve with two-fold serial dilutions with the highest standard of 500 pg/mL.

### Real-time PCR

TM cells were untreated, or infected with CMV AD169 at MOI of 1 and/or stimulated with recombinant active TGF-β1 at 15 ng/ml (0.6 nM) and/or treated with losartan (0.1 uM, 1 μM, 10 uM) for 48 hours [13]. Cells were washed, lysed and total RNA extracted using an RNeasy Mini Kit (Qiagen, Valencia, CA, USA). The cDNAs were amplified and quantified (PrimeScript RT reagent Kit, TaKaRa Bio, Kusatsu, Japan). The relative expression levels of mRNA were determined using a Roche Diagnostics LightCycler 2.0 Real-Time PCR System (Roche, Mannheim, Germany). The sequences of the real-time PCR primer pairs are shown in Table 1. To ensure equal loading and amplification, all products were normalized to a β-actin transcript as an internal control.

**Table 1:** Sequence for forward and reverse primer sets used for real-time PCR.

Amplification	Forward primer	Reverse primer
LOX	5’-CGACCCTTACAACCCCTACA-3’	5’-AAGTAGCCAGTGCCGTATCC-3’
α-SMA	5′ -GACAATGGCTCTGGGCTCTGTAA-3′	5′ -CTGTGCTTCGTCACCCACGTA-3′
collagen1A	5′ -GGAATGAAGGGACACAGAGG-3′	5′ -TAGCACCATCATTTCCACGA-3′
CTGF	5′ -CTCCTGCAGGCTAGAGAAGC-3′	5′ -GATGCACTTTTTGCCCTTCTT-3′
fibronectin	5′ -CTGGCCGAAAATACATTGTAA-3′	5′ -CCACAGTCGGGTCAGGAG-3′
RhoA	5’ -CGTTAGTCCACGGTCTGGTC-3’	5’-GCCATTGCTCAGGCAACGAA-3’
PDGF-B	5’ -TGATCTCCAACGCCTGCT-3’	5’-TCATGTTCAGGTCCAACTCG-3’
MCP-1	5’ -CTGAAGCTCGTACTCTC-3’	5’-CTTGGGTTGTGGAGTGAG-3’
β-actin	5′ -GTCCACCTTCCAGCAGATGT-3′	5′ -AAAGCCATGCCAATCTCATC-3′

LOX: lysyl oxidase; α -SMA: smooth muscle actin; CTGF: connective tissue growth factor; TGF: transforming growth factor; PDGF: platelet derived growth factor; MCP: Monocyte chemoattractant protein.

### Immunofluorescence staining

Confluent cells seeded on 6-well plates were infected with CMV AD169 at the indicated MOIs of 1 or 0.1 and fixed at 1 dpi, then the immediate-early (IE) antigens at 1 dpi were analyzed by immunofluorescence imaging as described previously [16]. After cells were fixed, treated, and incubated with a blocking buffer, the cells were immunolabeled with anti-CMV IE antibody (1:50) overnight at 4°C and stained with a secondary goat anti-mouse antibody for 1 h. Rhodamine phalloidin (Invitrogen) was used to visualize stress fiber structures. Images were obtained using inverted fluorescence microscopy (IX83; Olympus, Tokyo, Japan).

### Statistical analysis

For comparisons between two groups, the independent *t*-test was used. For comparison of results among three groups, one-way analysis of variance was performed. Experiments were performed in triplicate and representative results are reported. A value of *P* < 0.05 was considered to indicate statistical significance.

## Results

### The effects of dexamethasone, losartan, and ganciclovir on CMV viral replication

**Fig 1:**
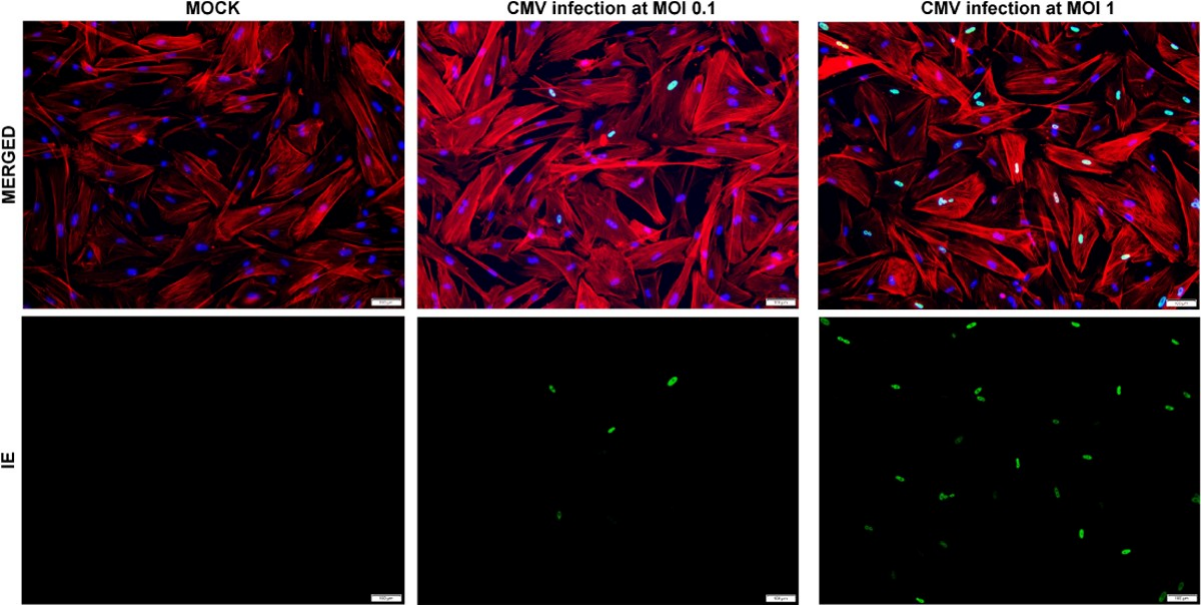
Primary cultured human trabecular meshwork cell (TM) inoculated with human cytomegalovirus strain AD169. **Normal uninfected TM cells and TM cells at a multiplicity of infection of 0.1 or 1.** To confirm the infectivity of CMV AD 169, the infected cells were immunolabeled with an anti-IE1 antibody at 1 day post infection (dpi). IE, immediate early (green signal), and stress fibers with a Rhodamine Phalloidin (red signals). Bar = 200 μm.

**Fig 2:**
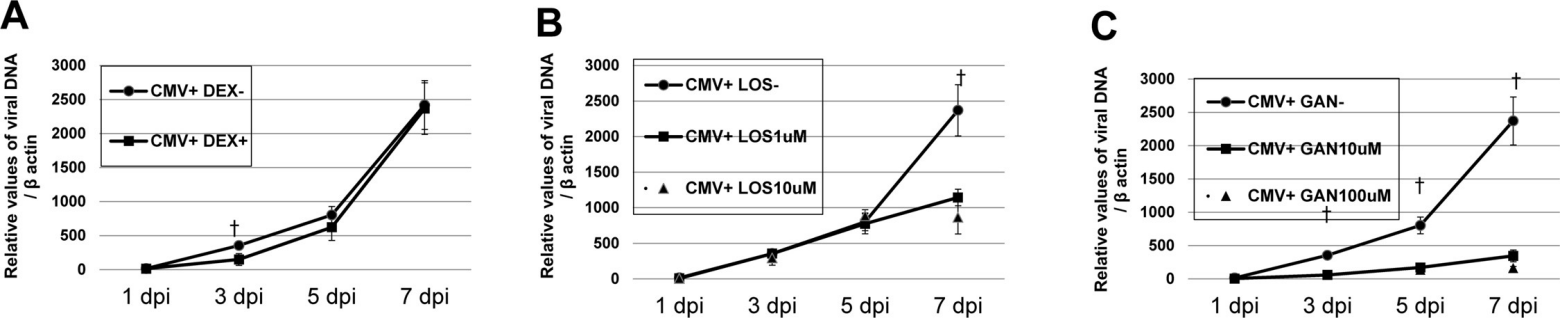
**Viral DNA accumulation after human cytomegalovirus infection and dexamethasone (A) losartan (B), and ganciclovir treatment (C) in human trabecular meshwork (TM) cells.** Cells were infected with CMV AD169 at a high multiplicity of infection (MOI; 1). Cells were harvested at 1, 3, 5, and 7 day after infection (dpi) and viral DNA was extracted from cells and processed for qPCR analysis of viral DNA accumulation (UL26). Real-time PCR with β -actin primers was performed to serve as an internal control for input DNA. Data are the averages of three independent DNA samples from the infected cells. Values are mean ± standard error.

After CMV infection in TM cells, the IE antigen was detected at 1 dpi, at high and low MOI (Fig 1) (also at 3 and 5 dpi, data in S1 and S2 Figs). The amount of viral DNA also increased at 1, 3, 5, and 7 dpi in human TM cells after CMV AD169 infection (solid line, Fig 2A). Dexamethasone treatment did not have significant effect on the CMV copy numbers at 1, 5, and 7 dpi, except at 3 dpi (*P* < 0.05). Losartan treatment significantly decreased CMV copy number only at 7 dpi (*P* < 0.05, Fig 2B). As expected, ganciclovir treatment significantly decreased the viral copy number at 3, 5, and 7 dpi (*P* < 0.05; Fig 2C).

### The effects of dexamethasone, losartan, and ganciclovir on viability of TM cells

**Fig 3:**
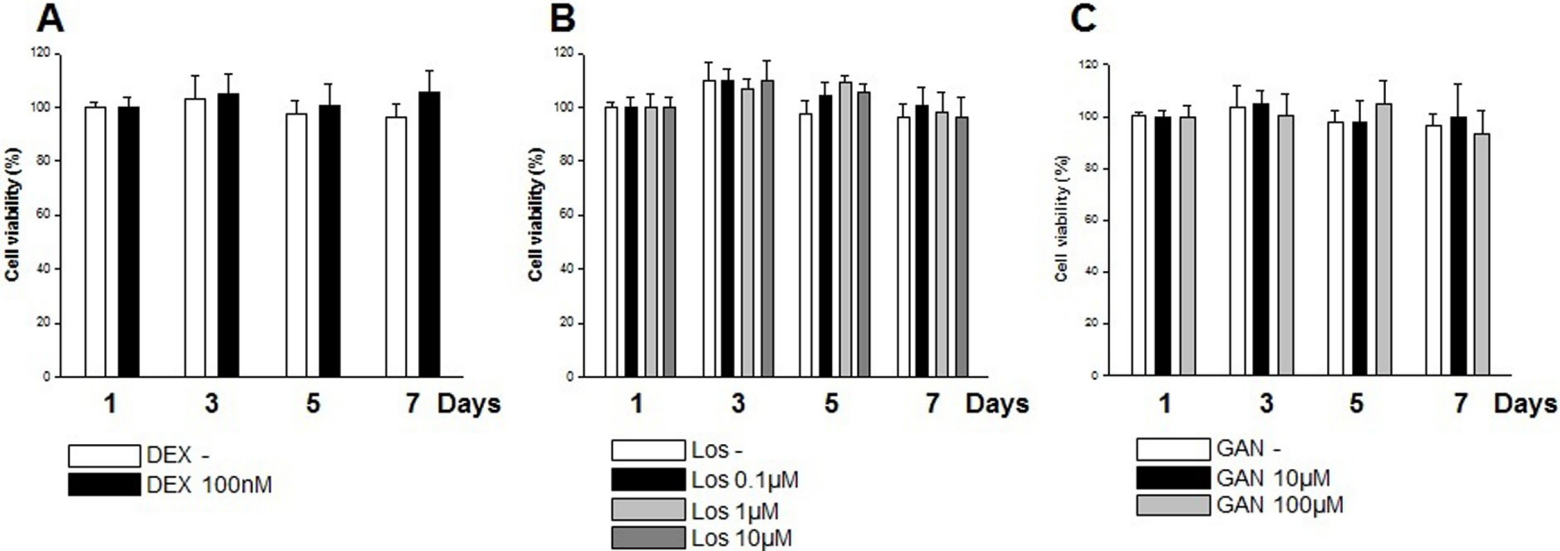
**Effects of dexamethasone (A) losartan (B), and ganciclovir treatment (C) on viabilities of cultured TM cells.** Viabilities of TM cells evaluated using MTS assay were not changed by any concentration of treatment. Each value is expressed the mean ± SE.

The effects of dexamethasone, losartan, and ganciclovir on viability of TM cells were evaluated with MTS assay. Human TM cells were treated with various concentrations of dexamethasone (0, 100 μM) or losartan (0, 0.1 μM, 1 μM, 10 μM), or ganciclovir (0 μM, 10 μM, 100 μM) for 1, 3, 5, 7 days respectively. Cell viability of TM cells was not changed by any of the examined concentrations of agents across the experiments (Fig 3).

### CMV-induced secretion of TGF-β1 quantitated by ELISA

**Fig 4:**
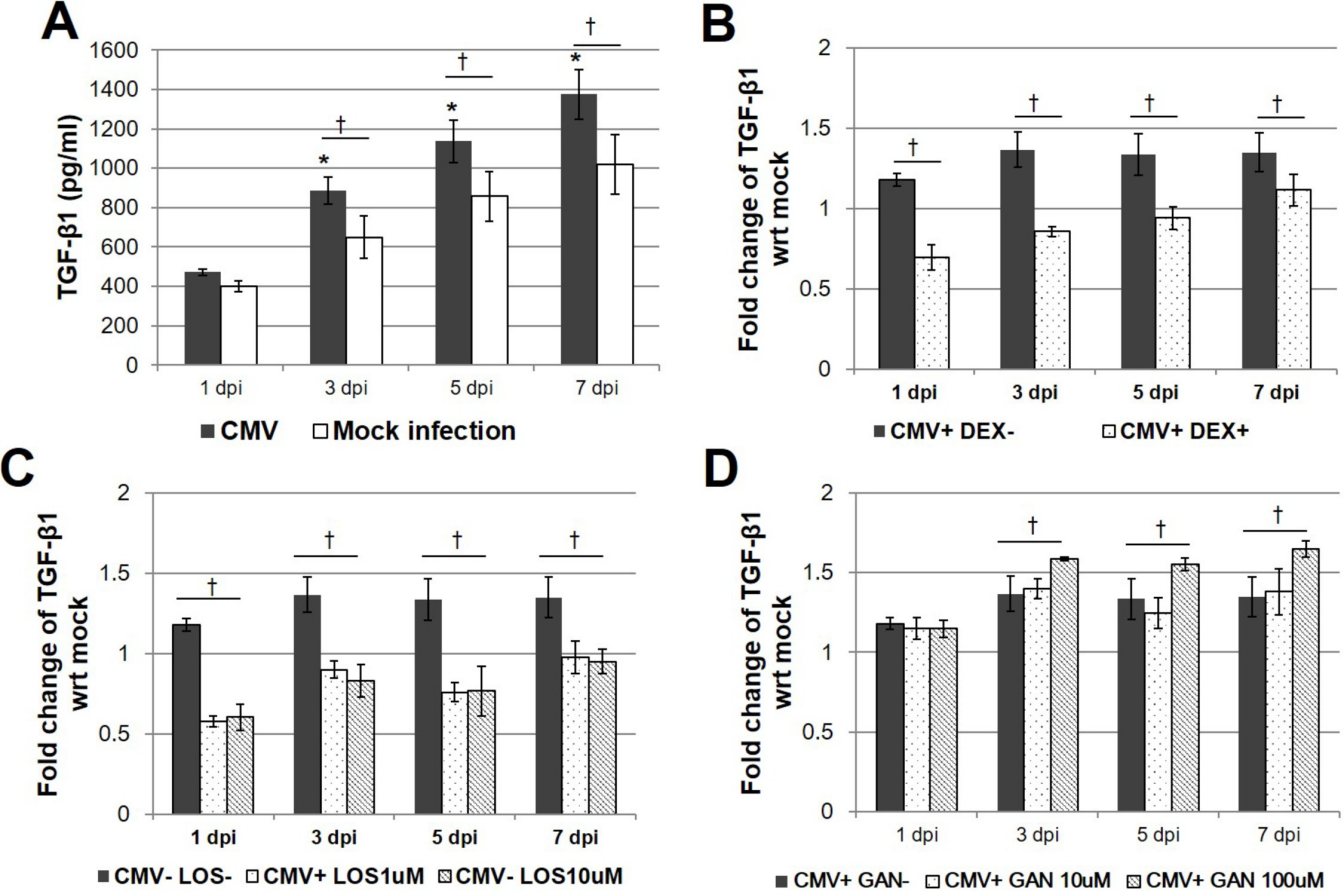
**TGF-β1 production after human cytomegalovirus infection (A) and dexamethasone (B) losartan (C), and ganciclovir treatment (D) in human trabecular meshwork (TM) cells.**. Cells were infected with CMV AD169 at a high multiplicity of infection (MOI; 1). Cells were harvested at 1, 3, 5, and 7 days after infection (dpi). Supernatants were assayed for total TGF-β1 production using a TGF-β1 responsive luciferase bioassay. (A) Expression of TGF-β1 was increased 1 dpi (*P < 0.05 vs. TGF-β expression 1 dpi; ^†^*P* < 0.05 vs. TGF-β expression of the Mock infection). (B) Treatment with DEX significantly decreased the production of TGF-β1 5 dpi (*P < 0.05 for TGF-β1 expression of CMV infection without DEX, CMV infection with DEX, and DEX treatment alone). Results are expressed as the mean ± standard deviation of three different experiments. DEX, dexamethasone.

To identify the effect of CMV infection in outflow pathway, the expression of TGF- β1 were measured. When the CMV-induced secretion of TGF-β1 was examined by ELISA, significant increases in the concentration of TGF-β1 in the CMV-infected TM cells at MOI of 1 were detected at MOI of 3, 5, and 7 dpi (*P* < 0.001; Fig 4A). Treatment with dexamethasone significantly decreased the secretion of TGF-β1 at 1, 3, 5, and 7 dpi (Fig 4B). Treatment with losartan (1 and 10 μM) also significantly decreased the levels of TGF-β1 at 1, 3, 5, and 7 dpi (Fig 4C). However, treatment with 10 μM ganciclovir did not affect the TGF-β1 levels throughout the observation period. There was a significant increase in TGF-β1 levels with 100 μM ganciclovir treatment (Fig 4D).

### Effect of losartan on TM cell fibrogenic activity

**Fig 5:**
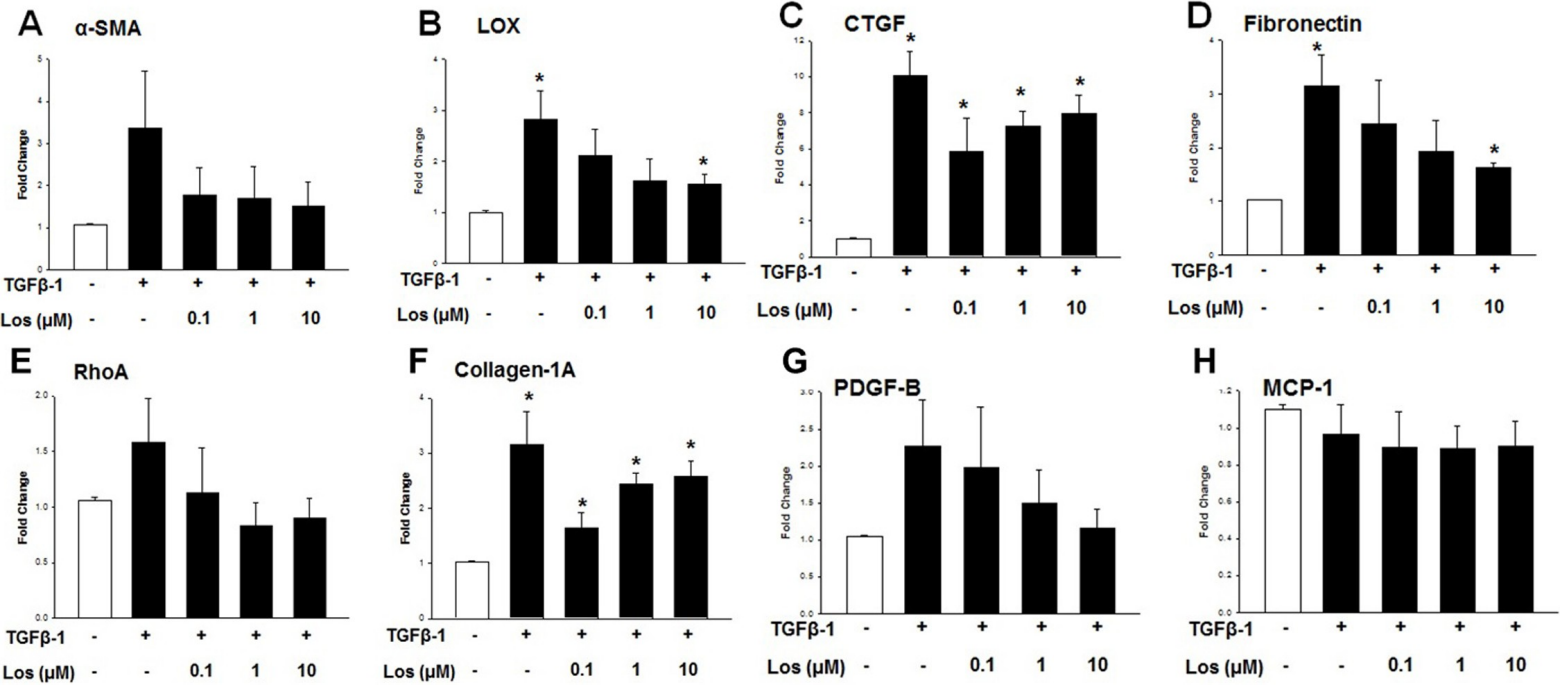
Quantitative determination of mRNA expression levels according to losartan treatment with or without TGF- β1 for 48 hours. (A) expression of α-smooth muscle actin (SMA), (B) lysyl oxidase (LOX), (C) connective tissue growth factor (CTGF), (D) fibronectin, (E) RhoA, (F) collagen-1A, (G) PDGF-B, and (H) MCP-1 (*P < 0.05 vs. transcripts from the unstimulated Mock infection).

Based on above described observation on the role of CMV infection in TGF-β1 induction, we tested the effects of losartan on the expression profile of fibrogenic markers induced by TGF- β1 in human TM cells. Serum-starved TM cells treated with TGF-β1 along with the presence of losartan (0.1 μM, 1 μM, 10 μM) for 48 hours showed marked decrease in the expression of α-smooth muscle actin (SMA), lysyl oxidase (LOX), connective tissue growth factor (CTGF), fibronectin and collagen-1A, compared with TGF-β1 alone (Fig 5). Especially, the losartan treatment significantly decreased the expression of LOX and fibronectin in a dose-dependent manner (Fig 5B and 5D).

### Expression of fibrogenic and inflammatory molecules according to CMV infection with or without treatment with TGF-β1 and/or losartan using real-time PCR

**Fig 6:**
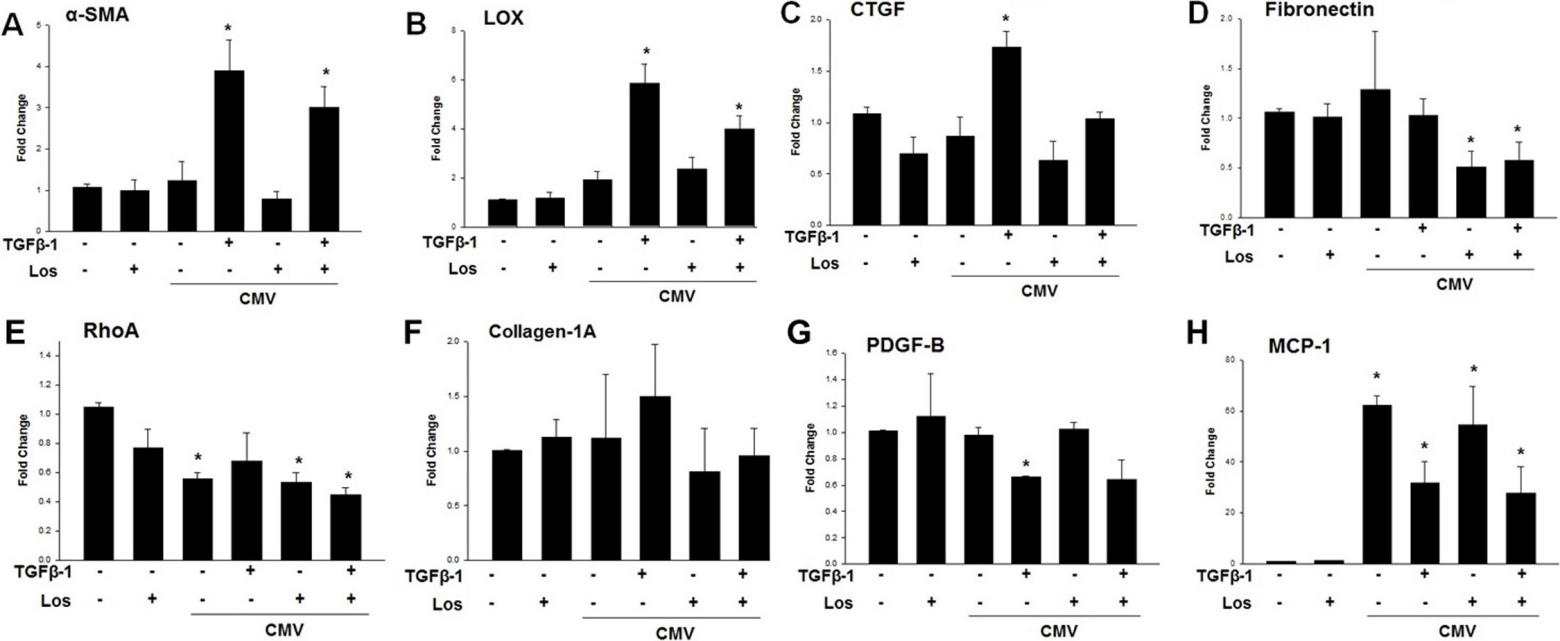
Quantitative determination of mRNA expression levels according to CMV AD169 infection with or without TGF- β1 and/or losartan (0.1 μM) 48 hours post infection. (A) expression of α-smooth muscle actin (SMA), (B) lysyl oxidase (LOX), (C) connective tissue growth factor (CTGF), (D) fibronectin, (E) RhoA, (F) collagen-1A, (G) PDGF-B, and (H) MCP-1 ((*P < 0.05 vs. transcripts from the unstimulated Mock infection).

To ascertain the effect of losartan on CMV-infected TM cells, CMV-infected TM cells were stimulated with or without TGF-β1 and/or losartan to investigate their effects on the transcripts of potential fibrogenic molecules. Compared with unstimulated mock infection, CMV-infected TM cells stimulated by TGF-β1 showed significantly enhanced expression of α-SMA, LOX, CTGF, and fibronectin, which were decreased with additional treatment with 1 μM losartan (Fig 6A–6D).

Compared with unstimulated mock infection, expression of RhoA and platelet-derived growth factor (PDGF)-B were significantly decreased after CMV infection. However, expression of monocyte chemoattractant protein (MCP)-1 significantly increased over 60-fold after CMV infection, and additional TGF-β1 treatment of CMV-infected TM cells decreased the expression of MCP-1 (Fig 6E–6H).

## Discussion

CMV is an important cause of retinitis in patients who have impaired T cell function, as a result of transplantation, AIDS, or immunosuppressive treatment. However, in the last 10 years, the involvement of CMV has been increasingly recognized in hypertensive anterior uveitis in immunocompetent, in which CMV DNA is detected in aqueous by PCR amplification [1]. The clinical features of CMV-induced anterior uveitis include Posner-Schlossmann syndrome, chronic anterior uveitis, and corneal endotheliitis. Especially, younger patients in their third to fifth decades with CMV anterior uveitis usually present with Posner-Schlossmann syndrome, which is characterized by minimal inflammation in anterior chamber, with minimal or no circumciliary injection, a single or a few central keratic precipitates [27].

TGF-β1 is known to be induced by CMV infection in various cells, mediating pro-fibrotic changes after CMV infection [13–15]. This study also confirmed that the secretion of TGF-β1 was significantly increased from 3 to 7 dpi in infected cells compared with mock-infected cells (Fig 4A), and the IE protein was found from 1 dpi (Fig 1) in human TM cells. These results were consistent with the previous finding that the TGF-β1 promoter is activated by the IE protein, and that enhanced TGF-β1 secretion is an early response to CMV infection [14].

Systemic RAS has an obligatory role in the regulation of blood pressure homeostasis, systemic fluid volume, and electrolyte balance. The circulatory RAS system is activated by renin, which cleaves angiotensinogen to form angiotensin I (Ang-I), which is then converted to angiotensin II (Ang-II) by the angiotensin-converting enzyme [28]. Ang-II regulates fibrosis, inflammation, and proliferation as well as vasoconstriction and electrolyte homeostasis through the activation of AT1R. The inhibition of Ang-II is effective in the prevention of cardiac remodeling and fibrotic conditions in renal and liver tissues [29]. Local RAS has been found in various extra-renal tissues, including the thymus, adrenal glands, and ocular tissues [22], and all recognized RAS components have been detected in human ocular tissue [20, 30]. The inhibition of Ang-II therefore represents a potential therapeutic target of ECM remodeling in ocular tissue.

The pro-fibrotic cascade by Ang-II is thought to be mediated by increased TGF-β, as reported by Kagami, et al. [23], and Ang-II treatment of rat mesangial cells in culture increased the levels of TGF-β. With the induction of TGF-β, Ang-II treatment of rat mesangial cells also enhanced the expression of matrix components biglycan, fibronectin, and collagen type 1, which was prevented by a competitive inhibitor of Ang-II [23]. Miguel-Carrasco et al. [31] showed that losartan metabolites showed anti-fibrotic effect by blockade of CTGF-induced LOX in fibroblast. Consistent with these results, in this study, losartan attenuated the expression of various fibrogenic molecules induced by TGF-β1, especially of LOX and fibronectin in a dose-dependent manner (Fig 5B and 5D). In this study, replication of viral DNA was reduced with losartan treatment at 7 dpi, as well as treatment with the anti-viral agent ganciclovir (Fig 2B and 2C). No additional cytotoxicity was noted with the treatment of dexamethasone, losartan, and ganciclovir (Fig 3). However, no dose-dependent response was noted with the application of losartan. In this regard, losartan appears to have a direct anti-fibrotic effect on human TM cells rather than working through the etiology of fibrosis of TM cells.

Interestingly, our study showed that the enhanced expression of TGF-β1 induced by CMV infection was significantly decreased by treatment with 1 μM losartan, which is a selective AT1R inhibitor (Fig 4C). Notably, we found that the expression of fibrogenic molecules such as α-SMA and CTGF was significantly elevated during CMV infection when stimulated by TGF-β1, which is present in the aqueous humor and functions in the normal physiology of the eye. The enhanced expression of fibrotic molecules (α-SMA, CTGF, LOX and fibronectin) was significantly reduced by treatment with losartan (Fig 6). TGF-β1 is one of the downstream molecules of Ang-II [19]. Therefore, inhibition of Ang-II would interrupt TGF-β1 following a pro-fibrogenic cascade during CMV infection in TM cells. Therefore, treatment with losartan may have therapeutic potential in the treatment of CMV anterior uveitis.

There is a possibility that the virus uses the RAS system in its infection. Virus-induced cardiac myopathy was decreased in the AT1R knockout mouse, suggesting that the AT1R signal is obligatory for the development of virus-induced myocardial injury [32]. It has also been reported that CMV infection stimulates the expression of renin and Ang-II in both kidney cells and the ECM in a dose-dependent manner [33]. The anti-viral properties of losartan have been reported by Gardner et al. [34], who showed that treatment with losartan caused a dose-dependent decrease in HSV-2 infectivity in cultured cardiac and Vero cells, suggesting that losartan prevents viral release from the cells. Further studies are needed to investigate potential anti-viral effect of losartan in CMV infection.

Our study also showed that CMV induced a 60-fold increase in the expression of MCP-1, which was decreased with TGF-β1 co-treatment (Fig 6H), whereas no significant changes in PDGF-B were noted (Fig 6G). It is known that CMV infection causes increased expression of several proinflammatory cytokines such as interleukin-6, tumor necrosis factor-α, and MCP-1 in serum in a CMV-infected animal model [33]. Importantly, MCP-1, which exhibits potent chemotactic activity in monocytes, is upregulated at an early stage of CMV infection [35]. In pancreatic cancer cells, Ang-II stimulates the expression of MCP-1 [36]. In a hyperuricemic nephropathy rat model, treatment with losartan decreased the expression of MCP-1 [37]. However, in our study, MCP-1 expression was not significantly affected by treatment with losartan. TGF-β1 is known to inhibit inflammation-medicated induction of MCP-1 in macrophages [38]. It is possible that stimulation with TGF-β1 greatly inhibited the expression of MCP-1, masking the effect of losartan on the expression of MCP-1. Further studies are therefore required to determine the effects of MCP-1 on the IOP regulatory mechanisms in CMV anterior uveitis.

Although there are many studies regarding the potential usage of RAS inhibitor as an ocular treatment, the clinical use of RAS inhibitor needs further studies on efficacy, pharmacokinetics, and safety. The application of systemic administration of losartan can impair the blood supply in the optic nerve head, potentially causing ischemic damage to retinal nerve fibers [20]. Considering that RAS components have also been identified in central structure of the eye, the local administration of RAS inhibitors including topical administration or intravitreal injection would be preferable.

Our study has limitations to be acknowledged. We used the laboratory strain AD169 throughout the experiment. Although AD169 is one of most widely used CMV strains, this is high passage laboratory strain, which has lost several virulence genes. The experiment was based on an *in vitro* infection model of cultured TM cells, rather than *in vivo* infection, which limits the clinical implication of the study. Further studies using animal model of CMV induced hypertensive uveitis are required.

In summary, CMV infection in human TM cells induced TGF-β1 as well as the pro-fibrotic cascade. The fibrotic changes induced by CMV infection were attenuated by treatment with losartan. Based on its effects on the modulation of fibrosis in TM cells, losartan may provide an effective treatment for patients with CMV-induced hypertensive anterior uveitis.

The English in this document has been checked by at least two professional editors, both native speakers of English. For a certificate, please see: http://www.textcheck.com/certificate/nVzMxW

## Funding

The authors wish to acknowledge the financial support of the National Research Foundation of Korea funded by the Korean government (No. NRF- 2019R1F1A1043806 and 2016R1A6A1A03010528).

## Supporting information

S1 Fig Primary cultured human trabecular meshwork cell (TM) inoculated with human cytomegalovirus strain AD169 at 3 day post infection (dpi). **Normal uninfected TM cells and TM cells at a multiplicity of infection of 0.1 or 1.** To confirm the infectivity of CMV AD 169, the infected cells were immunolabeled with an anti-IE1 antibody. IE, immediate early (green signal), and stress fibers with a Rhodamine Phalloidin (red signals). Bar = 200 μm.
(TIF)

S2 Fig Primary cultured human trabecular meshwork cell (TM) inoculated with human cytomegalovirus strain AD169 at 5 day post infection. **Normal uninfected TM cells and CMV-infected TM cells at a multiplicity of infection of 0.1 or 1.** To confirm the infectivity of CMV AD 169, the infected cells were immunolabeled with an anti-IE1 antibody. IE, immediate early (green signal), and stress fibers with a Rhodamine Phalloidin (red signals). Bar = 200 μm.
(TIF)

## References

[1] Chan NS, Chee SP. Demystifying viral anterior uveitis: a review. 2018 10.1111/ceo.13417 30345620

[2] Chee SP, Bacsal K, Jap A, Se-Thoe SY, Cheng CL, Tan BH. Corneal endotheliitis associated with evidence of cytomegalovirus infection. Ophthalmology. 2007;114(4):798–803. Epub 2007/01/09. 10.1016/j.ophtha.2006.07.057 . 17207531

[3] van Boxtel LA, van der Lelij A, van der Meer J, Los LI. Cytomegalovirus as a cause of anterior uveitis in immunocompetent patients. Ophthalmology. 2007;114(7):1358–62. Epub 2007/02/14. 10.1016/j.ophtha.2006.09.035 . 17296229

[4] Miyanaga M, Sugita S, Shimizu N, Morio T, Miyata K, Maruyama K, et al A significant association of viral loads with corneal endothelial cell damage in cytomegalovirus anterior uveitis. The British journal of ophthalmology. 2010;94(3):336–40. Epub 2009/09/08. 10.1136/bjo.2008.156422 . 19734135

[5] Kandori M, Miyazaki D, Yakura K, Komatsu N, Touge C, Ishikura R, et al Relationship between the number of cytomegalovirus in anterior chamber and severity of anterior segment inflammation. Japanese journal of ophthalmology. 2013;57(6):497–502. Epub 2013/08/10. 10.1007/s10384-013-0268-2 . 23928983

[6] Jap A, Sivakumar M, Chee SP. Is Posner Schlossman syndrome benign? Ophthalmology. 2001;108(5):913–8. Epub 2001/04/26. . 11320022 10.1016/s0161-6420(01)00551-6

[7] Su CC, Hu FR, Wang TH, Huang JY, Yeh PT, Lin CP, et al Clinical outcomes in cytomegalovirus-positive Posner-Schlossman syndrome patients treated with topical ganciclovir therapy. American journal of ophthalmology. 2014;158(5):1024-31.e2. Epub 2014/08/16. 10.1016/j.ajo.2014.08.007 . 25124264

[8] de Schryver I, Rozenberg F, Cassoux N, Michelson S, Kestelyn P, Lehoang P, et al Diagnosis and treatment of cytomegalovirus iridocyclitis without retinal necrosis. The British journal of ophthalmology. 2006;90(7):852–5. Epub 2006/04/07. 10.1136/bjo.2005.086546 16597667 PMC1857164

[9] Martin WJ 2nd, McDougall JC. Cytomegalovirus infection with idiopathic pulmonary fibrosis. Diagnosis suggested by bronchoalveolar lavage. Chest. 1983;84(4):500–2. Epub 1983/10/01. 10.1378/chest.84.4.500 . 6311490

[10] van Dam JG, Li F, Yin M, You XM, Grauls G, Steinhoff G, et al Effects of cytomegalovirus infection and prolonged cold ischemia on chronic rejection of rat renal allografts. Transplant international: official journal of the European Society for Organ Transplantation. 2000;13(1):54–63. Epub 2000/04/01. . 10743691 10.1007/s001470050009

[11] Li Y, Gao J, Wang G, Fei G. Latent cytomegalovirus infection exacerbates experimental pulmonary fibrosis by activating TGF-beta1. Molecular medicine reports. 2016;14(2):1297–301. Epub 2016/06/10. 10.3892/mmr.2016.5366 . 27279470

[12] Helantera I, Loginov R, Koskinen P, Tornroth T, Gronhagen-Riska C, Lautenschlager I. Persistent cytomegalovirus infection is associated with increased expression of TGF-beta1, PDGF-AA and ICAM-1 and arterial intimal thickening in kidney allografts. Nephrology, dialysis, transplantation: official publication of the European Dialysis and Transplant Association—European Renal Association. 2005;20(4):790–6. Epub 2005/02/18. 10.1093/ndt/gfh714 . 15716293

[13] Shimamura M, Murphy-Ullrich JE, Britt WJ. Human cytomegalovirus induces TGF-beta1 activation in renal tubular epithelial cells after epithelial-to-mesenchymal transition. PLoS pathogens. 2010;6(11):e1001170 Epub 2010/11/17. 10.1371/journal.ppat.1001170 21079788 PMC2973835

[14] Michelson S, Alcami J, Kim SJ, Danielpour D, Bachelerie F, Picard L, et al Human cytomegalovirus infection induces transcription and secretion of transforming growth factor beta 1. Journal of virology. 1994;68(9):5730–7. Epub 1994/09/01. 8057454 10.1128/jvi.68.9.5730-5737.1994 PMC236976

[15] Kwon YJ, Kim DJ, Kim JH, Park CG, Cha CY, Hwang ES. Human cytomegalovirus (HCMV) infection in osteosarcoma cell line suppresses GM-CSF production by induction of TGF-beta. Microbiology and immunology. 2004;48(3):195–9. Epub 2004/03/20. . 15031532 10.1111/j.1348-0421.2004.tb03505.x

[16] Choi JA, Kim JE, Noh SJ, Kyoung Kim E, Park CK, Paik SY. Enhanced cytomegalovirus infection in human trabecular meshwork cells and its implication in glaucoma pathogenesis. Scientific reports. 2017;7:43349 Epub 2017/02/28. 10.1038/srep43349 28240260 PMC5327388

[17] Vranka JA, Kelley MJ, Acott TS, Keller KE. Extracellular matrix in the trabecular meshwork: intraocular pressure regulation and dysregulation in glaucoma. Experimental eye research. 2015;133:112–25. Epub 2015/03/31. 10.1016/j.exer.2014.07.014 25819459 PMC4379427

[18] Prendes MA, Harris A, Wirostko BM, Gerber AL, Siesky B. The role of transforming growth factor beta in glaucoma and the therapeutic implications. The British journal of ophthalmology. 2013;97(6):680–6. Epub 2013/01/17. 10.1136/bjophthalmol-2011-301132 . 23322881

[19] Agarwal P, Agarwal R. Trabecular meshwork ECM remodeling in glaucoma: could RAS be a target? Expert opinion on therapeutic targets. 2018;22(7):629–38. Epub 2018/06/09. 10.1080/14728222.2018.1486822 . 29883239

[20] Holappa M, Vapaatalo H, Vaajanen A. Many Faces of Renin-angiotensin System—Focus on Eye. The open ophthalmology journal. 2017;11:122–42. Epub 2017/08/02. 10.2174/1874364101711010122 28761566 PMC5510558

[21] Choudhary R, Kapoor MS, Singh A, Bodakhe SH. Therapeutic targets of renin-angiotensin system in ocular disorders. Journal of current ophthalmology. 2017;29(1):7–16. Epub 2017/04/04. 10.1016/j.joco.2016.09.009 28367520 PMC5362395

[22] Kurihara T, Ozawa Y, Ishida S, Okano H, Tsubota K. Renin-Angiotensin system hyperactivation can induce inflammation and retinal neural dysfunction. International journal of inflammation. 2012;2012:581695 Epub 2012/04/27. 10.1155/2012/581695 22536545 PMC3321303

[23] Kagami S, Border WA, Miller DE, Noble NA. Angiotensin II stimulates extracellular matrix protein synthesis through induction of transforming growth factor-beta expression in rat glomerular mesangial cells. The Journal of clinical investigation. 1994;93(6):2431–7. Epub 1994/06/01. 10.1172/JCI117251 8200978 PMC294451

[24] Stamer WD, Seftor RE, Williams SK, Samaha HA, Snyder RW. Isolation and culture of human trabecular meshwork cells by extracellular matrix digestion. Current eye research. 1995;14(7):611–7. Epub 1995/07/01. . 7587308 10.3109/02713689508998409

[25] Diskin S, Chen WS, Cao Z, Gyawali S, Gong H, Soza A, et al Galectin-8 promotes cytoskeletal rearrangement in trabecular meshwork cells through activation of Rho signaling. PloS one. 2012;7(9):e44400 Epub 2012/09/14. 10.1371/journal.pone.0044400 22973445 PMC3433423

[26] McArdle J, Moorman NJ, Munger J. HCMV targets the metabolic stress response through activation of AMPK whose activity is important for viral replication. PLoS pathogens. 2012;8(1):e1002502 Epub 2012/02/01. 10.1371/journal.ppat.1002502 22291597 PMC3266935

[27] Teoh SB, Thean L, Koay E. Cytomegalovirus in aetiology of Posner-Schlossman syndrome: evidence from quantitative polymerase chain reaction. Eye (London, England). 2005;19(12):1338–40. Epub 2004/11/16. 10.1038/sj.eye.6701757 . 15543169

[28] White AJ, Cheruvu SC, Sarris M, Liyanage SS, Lumbers E, Chui J, et al Expression of classical components of the renin-angiotensin system in the human eye. Journal of the renin-angiotensin-aldosterone system: JRAAS. 2015;16(1):59–66. Epub 2014/10/08. 10.1177/1470320314549791 . 25287897

[29] Salama ZA, Sadek A, Abdelhady AM, Darweesh SK, Morsy SA, Esmat G. Losartan may inhibit the progression of liver fibrosis in chronic HCV patients. Hepatobiliary surgery and nutrition. 2016;5(3):249–55. Epub 2016/06/09. 10.21037/hbsn.2016.02.06 27275467 PMC4876242

[30] Vaajanen A, Vapaatalo H. Local ocular renin-angiotensin system—a target for glaucoma therapy? Basic & clinical pharmacology & toxicology. 2011;109(4):217–24. Epub 2011/05/24. 10.1111/j.1742-7843.2011.00729.x . 21599836

[31] Miguel-Carrasco JL, Beaumont J, San Jose G, Moreno MU, Lopez B, Gonzalez A, et al Mechanisms underlying the cardiac antifibrotic effects of losartan metabolites. Scientific reports. 2017;7:41865 Epub 2017/02/06. 10.1038/srep41865 28157237 PMC5291109

[32] Yamamoto K, Shioi T, Uchiyama K, Miyamoto T, Sasayama S, Matsumori A. Attenuation of virus-induced myocardial injury by inhibition of the angiotensin II type 1 receptor signal and decreased nuclear factor-kappa B activation in knockout mice. Journal of the American College of Cardiology. 2003;42(11):2000–6. Epub 2003/12/10. . 14662266 10.1016/j.jacc.2003.07.021

[33] Cheng J, Ke Q, Jin Z, Wang H, Kocher O, Morgan JP, et al Cytomegalovirus infection causes an increase of arterial blood pressure. PLoS pathogens. 2009;5(5):e1000427 Epub 2009/05/14. 10.1371/journal.ppat.1000427 19436702 PMC2673691

[34] Gardner PL, Mbuy GN, Knabb MT. Effects of the angiotensin II receptor antagonist losartan on herpes simplex virus-type 2 infection of cultured vero and cardiac neonatal myocytes. Life sciences. 1994;55(4):283–9. Epub 1994/01/01. . 8028446 10.1016/0024-3205(94)00730-6

[35] Hamilton ST, Scott GM, Naing Z, Rawlinson WD. Human cytomegalovirus directly modulates expression of chemokine CCL2 (MCP-1) during viral replication. The Journal of general virology. 2013;94(Pt 11):2495–503. Epub 2013/08/14. 10.1099/vir.0.052878-0 . 23939977

[36] Chehl N, Gong Q, Chipitsyna G, Aziz T, Yeo CJ, Arafat HA. Angiotensin II regulates the expression of monocyte chemoattractant protein-1 in pancreatic cancer cells. Journal of gastrointestinal surgery: official journal of the Society for Surgery of the Alimentary Tract. 2009;13(12):2189–200. Epub 2009/10/10. 10.1007/s11605-009-1055-8 . 19816747

[37] Yu S, Ren Q, Wu W. Effects of losartan on expression of monocyte chemoattractant protein-1 (MCP-1) in hyperuricemic nephropathy rats. Journal of receptor and signal transduction research. 2015;35(5):458–61. Epub 2015/04/02. 10.3109/10799893.2015.1006332 . 25830624

[38] Feinberg MW, Shimizu K, Lebedeva M, Haspel R, Takayama K, Chen Z, et al Essential role for Smad3 in regulating MCP-1 expression and vascular inflammation. Circulation research. 2004;94(5):601–8. Epub 2004/01/31. 10.1161/01.RES.0000119170.70818.4F . 14752027

