# Correction: The effects of losartan on cytomegalovirus infection in human trabecular meshwork cells

**DOI:** 10.1371/journal.pone.0221693

**Published:** 2019-08-22

**Authors:** Jin A. Choi, Ju-Eun Kim, Hyun-hee Ju, Jiyoung Lee, Donghyun Jee, Chan Kee Park, Soon-young Paik

There are errors in the Funding statement. The correct Funding statement is as follows: The authors wish to acknowledge the financial support of the following grant: the development fund of Catholic Institute for Visual Science of 2019 and a National Research Foundation of Korea funded by the Korean government (No. NRF- 2019R1F1A1043806 and 2016R1A6A1A03010528).

## References

[1] Choi JA, Kim J-E, Ju H-h, Lee J, Jee D, Park CK, et al (2019) The effects of losartan on cytomegalovirus infection in human trabecular meshwork cells. PLoS ONE 14(6): e0218471 10.1371/journal.pone.0218471 31216320 PMC6584002

