# Supplementary figures and images for: The effects of losartan on cytomegalovirus infection in human trabecular meshwork cells
Source: PLoS One. 2019 Jun 19;14(6):e0218471. doi: 10.1371/journal.pone.0218471 (PMC6584002; doi:10.1371/journal.pone.0218471)

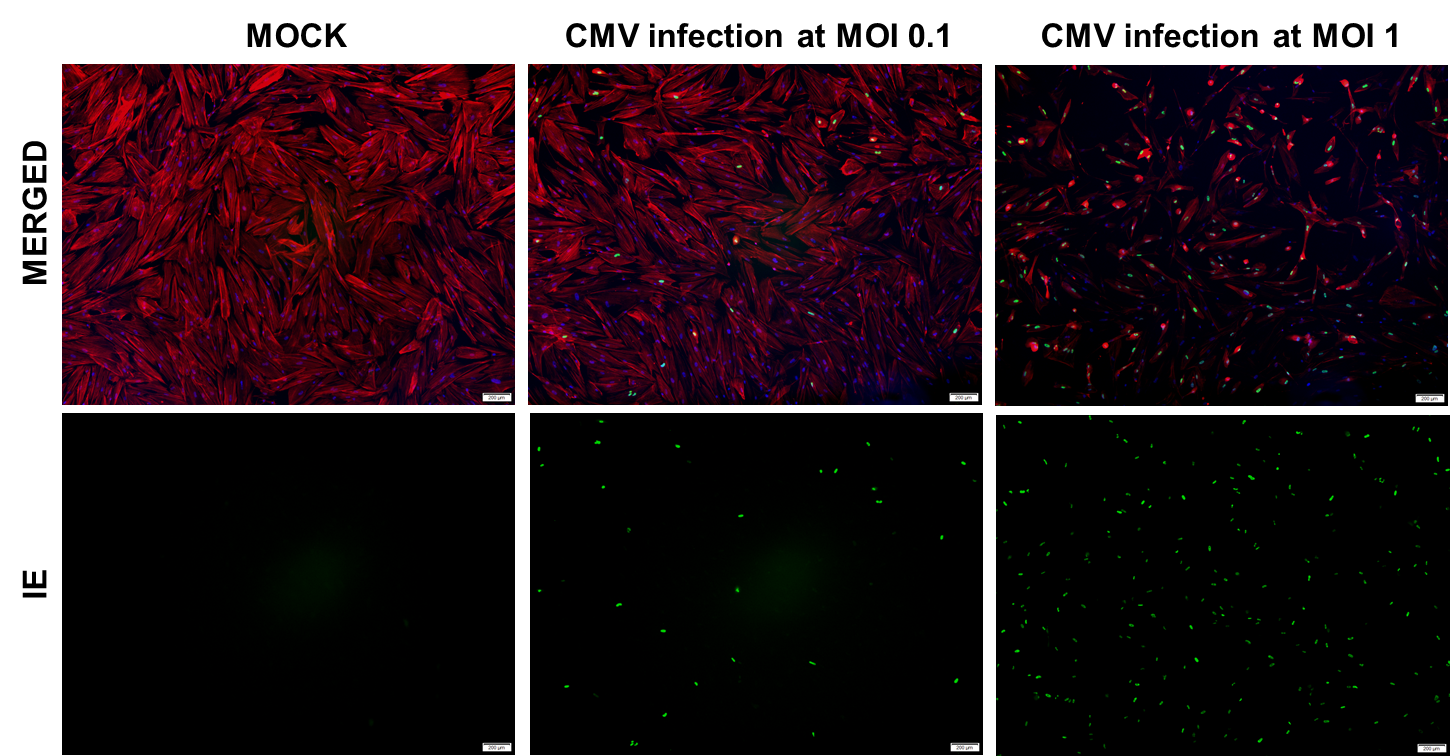

Supplement: S1 Fig — Normal uninfected TM cells and TM cells at a multiplicity of infection of 0.1 or 1. To confirm the infectivity of CMV AD 169, the infected cells were immunolabeled with an anti-IE1 antibody. IE, immediate early (green signal), and stress fibers with a Rhodamine Phalloidin (red signals). Bar = 200 μm. (TIF) [file pone.0218471.s001.tif]

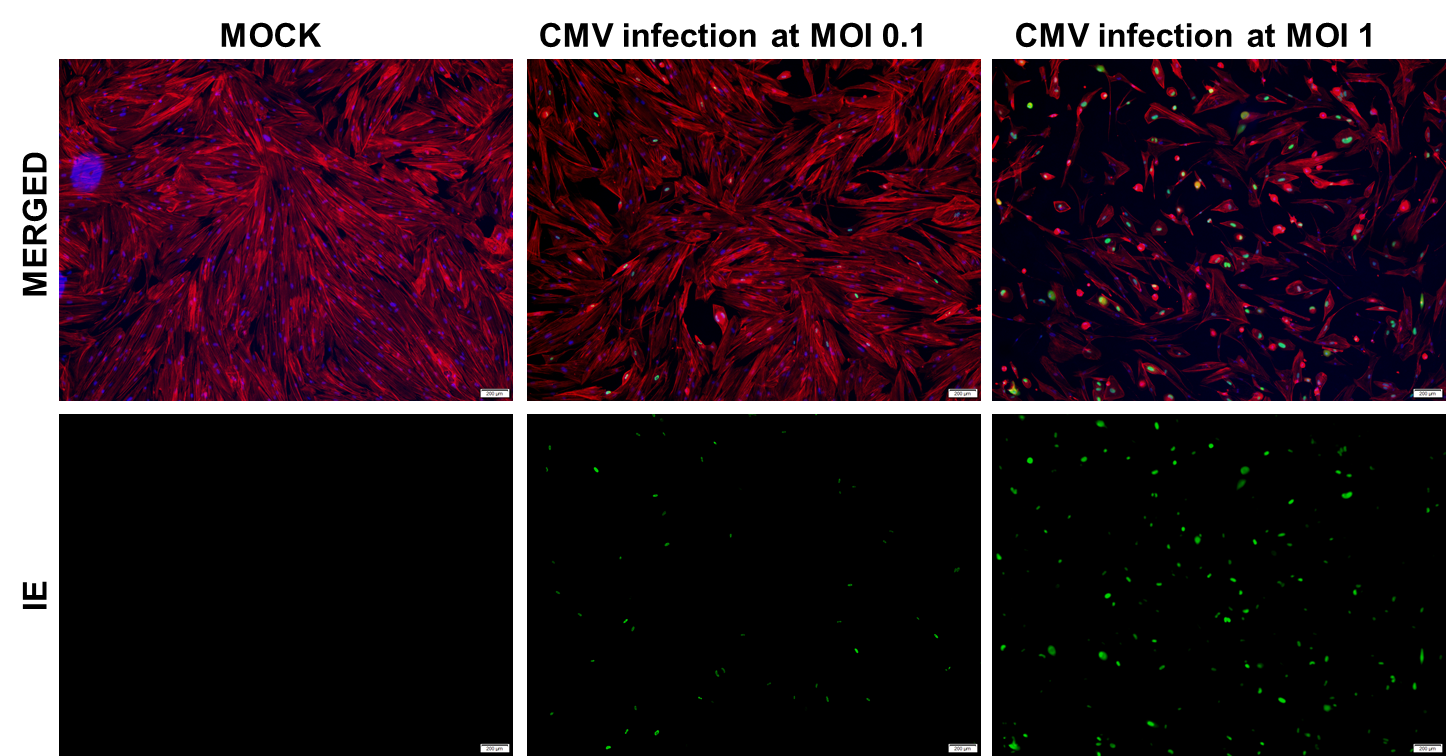

Supplement: S2 Fig — Normal uninfected TM cells and CMV-infected TM cells at a multiplicity of infection of 0.1 or 1. To confirm the infectivity of CMV AD 169, the infected cells were immunolabeled with an anti-IE1 antibody. IE, immediate early (green signal), and stress fibers with a Rhodamine Phalloidin (red signals). Bar = 200 μm. (TIF) [file pone.0218471.s002.tif]
